# The Effect of Individual Musculoskeletal Conditions on Depression: Updated Insights From an Irish Longitudinal Study on Aging

**DOI:** 10.3389/fmed.2021.697649

**Published:** 2021-08-26

**Authors:** Wenyi Jin, Zilin Liu, Yubiao Zhang, Zhifei Che, Mingyong Gao

**Affiliations:** ^1^Department of Orthopedics, Renmin Hospital of Wuhan University, Wuhan, China; ^2^Department of Urology, The First Affiliated Hospital of Hainan Medical University, Haikou, China; ^3^Department of Orthopedics, Jiangxi Provincial People's Hospital Affiliated to Nanchang University, Nanchang, China

**Keywords:** depression, late-life depression, musculoskeletal conditions, arthritis, pain

## Abstract

Few longitudinal studies have systematically investigated whether or how individual musculoskeletal conditions (IMCs) convey risks for negative psychological health outcomes, and approaches to assess such risk in the older population are lacking. In this Irish nationally representative longitudinal prospective study of 6,715 individuals aged 50 and above, machine learning algorithms and various models, including mediation models, were employed to elaborate the underlying mechanisms of IMCs leading to depression and to develop an IMC-induced negative psychological risk (IMCPR) classification approach. Resultantly, arthritis [odds ratio (95% confidence interval): 2.233 (1.700–2.927)], osteoporosis [1.681 (1.133–2.421)], and musculoskeletal chronic pain [MCP, 2.404 (1.838–3.151)] were found to increase the risk of depression after 2 years, while fracture and joint replacement did not. Interestingly, mediation models further demonstrated that arthritis *per se* did not increase the risk of depression; such risk was augmented only when arthritis-induced restrictions of activities (ARA) existed [proportion of mediation: 316.3% (ARA of usual), 213.3% (ARA of social and leisure), and 251.3% (ARA of sleep)]. The random forest algorithm attested that osteoarthritis, not rheumatoid arthritis, contributed the most to depressive symptoms. Moreover, bone mineral density was negatively associated with depressive symptoms. Systemic pain contributed the most to the increased risk of depression, followed by back, knee, hip, and foot pain (mean Gini-Index: 3.778, 2.442, 1.980, 1.438, and 0.879, respectively). Based on the aforementioned findings, the IMCPR classification approach was developed using an interpretable machine learning model, which stratifies participants into three grades. Among the IMCPR grades, patients with a grade of “severe” had higher odds of depression than those with a “mild” [odds ratio (95% confidence interval): 4.055 (2.907–5.498)] or “moderate” [3.584 (2.101–5.883)] grade. Females with a “severe” grade had higher odds of depression by 334.0% relative to those with a “mild” grade, while males had a relative risk of 258.4%. In conclusion, the present data provide systematic insights into the IMC-induced depression risk and updated the related clinical knowledge. Furthermore, the IMCPR classification approach could be used as an effective tool to evaluate this risk.

## Introduction

Human life expectancy worldwide has increased at an unprecedented rate during the last 200 years, and age can no longer be shaped by natural selection (1, 2). Population aging escalates the global burden of diseases, which makes explorations for reducing the length and severity of late-life morbidity a major goal (2). Psychiatric disorders contribute significantly to the global disease burden, and depression is the most common psychiatric disorder, which increases the disease burden (3, 4). Depression remains a major health problem in humans (5). Globally, over 300 million people and more than 25% of older people are compromised by depression, which severely disrupts psychosocial functioning and diminishes their quality of life (6–8). This disorder is deemed a leading cause of disability worldwide by the World Health Organization, and it often leads to tragic consequences, including suicide (9–11). Indeed, major depressive disorder as a prime cause of years lived with disability (YLD) contributes to 4.2% of total YLD worldwide (4); an existing depression longitudinal study also discovered that the overall long-term suicide risk varied from 5.6 to 6.8%, and the suicide risk of men with major depression reached as high as 20% (12). In addition, in the context of global turbulence (especially considering the recent outbreak of COVID-19), the socioeconomic burden of this debilitating disorder will continue to increase significantly (7, 13). From 2011 to 2030, the cumulative impact of mental-associated material expenditures on the global economy was estimated at US$16.3 trillion (13). This sobering and salient evidence indicates that it is extremely important to explore the factors that affect depression to control or prevent this disease.

Musculoskeletal conditions contribute even more to the global burden of disease among older people than do neurological and mental disorders (7.5 vs. 6.6%) (14); unfortunately, the two disorders are often entangled. Globally, over 1.3 billion people suffer from musculoskeletal disorders, which are a substantial contributor to non-fatal health loss (4). For example, osteoporosis escalates the propensity of fragility fractures (15), for which a fracture of the hip or spine carries a 12-month excess mortality rate of up to 20% (16). Osteoarthritis, as a major cause of disability worldwide, often results in pain, comorbidity, and mortality (17, 18). YLD was augmented by 54% between 1990 and 2015 owing to low back pain (19). The worst part is that musculoskeletal conditions confer mental disorders, including depression (20–23), which seriously threatens the quality of life of older people. However, how musculoskeletal conditions affect psychiatric health remains elusive.

To systematically interpret the roles and underlying mechanisms of musculoskeletal conditions on psychopathology risk, we relied on various models, including the mediation model and random forest algorithm (24–26), focusing on four classes of associations: arthritis-induced restriction of activities, arthritis subtypes, bone mineral density, and severe musculoskeletal chronic pain (MCP). After these works were completed, we re-examined the musculoskeletal conditions affecting psychopathological health and derived an IMCPR classification approach for negative psychological risk stratification of older people with musculoskeletal diseases.

## Materials and Methods

STROBE recommendations were employed to guide this reporting (27).

### Participants

We drew from The Irish Longitudinal Study on Aging (TILDA), a longitudinal study with two waves of population-representative data from Irish participants aged 50 years and above and those that were sampled using geographic-clusters-based RANSAM sampling system (28, 29). The study targeted individuals aged 50 and older living in the Republic of Ireland in residential accommodation (excluding those living in nursing homes and other institutions). Full informed consent was given by 8,504 participants, and Trinity College Dublin Research Ethics Committee issues the Ethical approval.

From October 2009 to February 2011, participants were contacted for a Wave 1 interview (30), and a Wave 2 interview was conducted from April 2012 to January 2013 (31). The response rate adjusted for eligibility is 62.0%. The cohorts for this study were recruited obeying the process shown in Supplementary Figure 1.

### Measures

Demographic data (i.e., gender and age, place of residence, smoking status, and marital status) were collected via a report at Wave 1.

Center for Epidemiological Studies Depression Scale (CES-D) was employed for depressive symptoms assessment. The CES-D is a 20-item scale assessing four major factors: depressed affect, absence of positive affect or anhedonia, somatic activity, or inactivity and interpersonal challenges (32). The response values are four-point Likert scales ranging from 0 to 3, yielding a total possible score of 60, with higher scores indicating greater depressive symptoms. When applied to the elder population, the CES-D scale works very well; an accumulated value of 16 and above was confirmed a sensitivity of 92% and a specificity of 87% for diagnosis of late-life depression (LLD) (33); the reliability coefficient was 0.85 to 0.91 for older people (34).

Individual musculoskeletal conditions were measured at Wave 1. Parameters collected includes arthritis (subtypes and degree of arthritis-induced restriction of activities), osteoporosis (*T*-score and stiffness), fracture, joint replacement, and MCP (most severe body part of MCP).

Osteoporosis increases the risk of fracture (35). An osteoporosis participant with a newly occurred fracture at Wave 2 (without fracture at Wave 1, but with fracture at Wave 2) was considered to be suffering from osteoporosis-related fracture.

Objective measures of physical health were collected in the fixed health assessment centers. These measures include *T*-score and stiffness (measured via heel ultrasound), triglyceride (TG), low-density lipoprotein cholesterol (LDL), high-density lipoprotein cholesterol (HDL), total cholesterol (CHOL), and body mass index (BMI).

Covariates were collected from theoretical, practical, or previous empirical evidence of association with musculoskeletal disorders or depression (36–39). Age, gender, place of residence, smoking status, marital status, chronic illnesses self-reported (i.e., an abnormal heart rhythm, congestive heart failure, angina, myocardial infarction, and diabetes), BMI, TG, LDL, HDL, CHOL, cognitive score, use of medication [i.e., benzodiazepines, antihypertensives (ATC C09, C08, C07, C03, C02), and anticholinergics (urological, antidepressant, other)], and alcohol were included.

### Statistical Analyses

Binomial logistic regression quantified the associations [i.e., odds ratios (OR)] between arthritis, osteoporosis, fracture, joint replacement, MCP, and probable LLD at baseline and Wave 2. Hosmer–Lemeshow test was conducted, and Nagelkerke *R*^2^ was subsequently calculated to evaluate the goodness of fit of models (40). Likelihood ratio tests assessed covariate significance (41).

For arthritis, arthritis-induced restriction of activities (ARA) was considered the potential mediators for mediation analysis (26). Kruskal–Wallis one-way ANOVA test (*H*-test, a pairwise comparison using Dwass–Steel–Crichtlow–Fligner test, and *p*-value adjustment using Benjamini and Hochberg method) revealed the potential difference of depressive symptoms among the different degrees of ARA (42). Stratified analyses were conducted to quantify associations between no arthritis, arthritis with/without ARA, and LLD. Gini Index calculated by random-forest algorithm (1,000 grew decision trees) quantified the contribution of subtypes (26).

For osteoporosis, osteoporosis-related fracture as a potential mediator participated in mediation analysis. Spearman's rank correlation coefficient assessed the correlations between bone mineral density (*T*-score and stiffness) and depressive symptoms. For MCP, the random-forest algorithm was performed to quantify contributions of the most severe body part of MCP.

Potential differences of depressive symptoms in musculoskeletal conditions were investigated by the Wilcoxon test, and *H*-test measured the gender-related potential difference.

IMCPR classification approach was constructed by decision tree regression referring to existing work (43), and musculoskeletal conditions associated with LLD were included for analysis. Associations between IMCPR grades and LLD were quantified (also considering gender-related associations).

R (version 3.6.3) was employed for statistical analyses. Packages used in this study are as follows: Hosmer–Lemeshow test (ResourceSelection package); likelihood ratio test (lmtest package); Wilcoxon test, Kruskal–Wallis one-way ANOVA test, Dwass–Steel–Crichtlow–Fligner test, Benjamini and Hochberg method used for *p*-value adjustment, and corresponding effect sizes calculation (ggstatsplot package); Random-Forest algorithm (randomForest package); and decision tree regression (rpart package).

## Results

### Demographics of the Participants at Baseline

The demographics of the Irish participants aged 50 and above are shown in Table 1. Among the musculoskeletal conditions, four (arthritis, osteoporosis, fracture, and MCP) had statistical significance between the no-depression and depression groups. Specifically, they were arthritis (*p* < 0.001, *V* = 0.083, 95% CI: 0.057–0.109, small effect size), osteoporosis (*p* < 0.001, *V* = 0.046, 95% CI: 0.014–0.073, small effect size), fracture (*p* = 0.007, *V* = 0.031, 95% CI: 0.004–0.057), and MCP (*p* < 0.001, *V* = 0.170, 95% CI: 0.146–0.195, small effect size).

**Table 1:** Demographics of Irish participants aged 50 and above on baseline.

**Parameters**		**No depression**	**Probable depression**	***p***	**Effect size (95% CI)**
		**(*n* = 6,099)**	**(*n* = 616)**		
**Age (years)**		62.00 (56.00–70.00)	59.00 (54.00–67.00)	<0.001	*r* = −0.056 (−0.080–0.039)
**Gender**	Male	2,882 (47.25)	205 (33.28)	<0.001	*V* = 0.080 (0.051–0.105)
	Female	3,217 (52.75)	411 (66.72)		
**Arthritis**	With	1,599 (26.22)	241 (39.12)	<0.001	*V* = 0.083 (0.057–0.109)
	Without	4,500 (73.78)	375 (60.88)		
**Osteoporosis**	With	578 (9.48)	89 (14.45)	<0.001	*V* = 0.046 (0.014–0.073)
	Without	5,521 (90.52)	527 (85.55)		
**Fracture**	With	801 (13.13)	105 (17.05)	0.007	*V* = 0.031 (0.004–0.057)
	Without	5,298 (86.87)	511 (82.95)		
**Joint replacements**	With	401 (6.57)	44 (7.14)	0.589	*V* = 0.000 (−0.026–0.014)
	Without	5,698 (93.43)	572 (92.86)		
**MCP**	With	2,001 (32.81)	376 (61.04)	<0.001	*V* = 0.170 (0.146–0.195)
	Without	4,098 (67.19)	240 (38.96)		

*Unless otherwise specified, all skewed continuous variables were described by “median (interquartile range)” and all categorical variables were described by “frequency [constituent ratio (%)].” CI, Confidence interval; r, Cohen's r; V, Cramer's V*.

### Preliminary Cross-Sectional and Longitudinal Results

**Figure 1:**
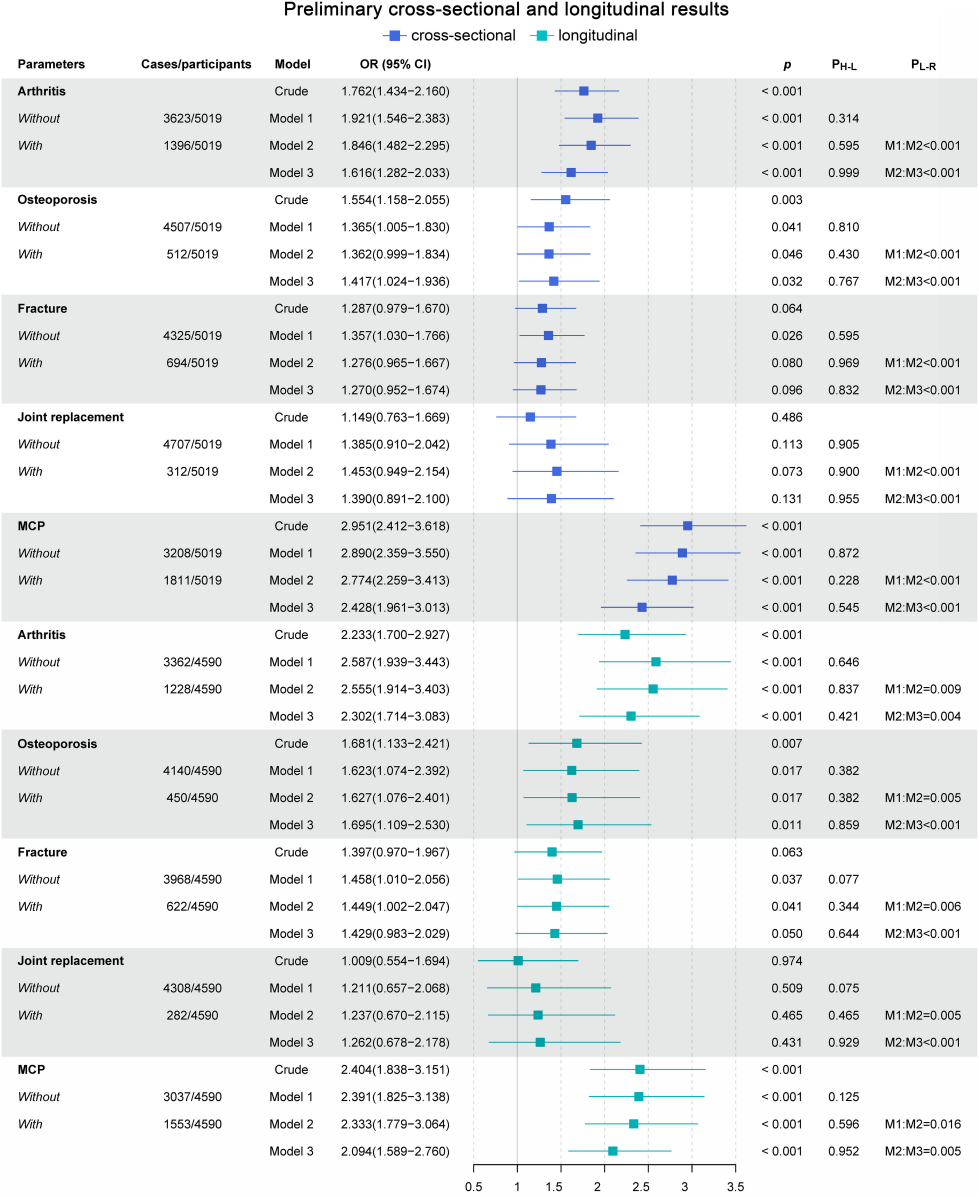
Preliminary cross-sectional and longitudinal results. Model 1 adjusted for gender and age. Model 2 adjusted for Model 1 and place of residence, smoking status, and marital status. Model 3 adjusted for Model 2 and an abnormal heart rhythm, congestive heart failure, angina, myocardial infarction, BMI, diabetes, TRIG, LDL, HDL, CHOL, cognitive score, use of medication [i.e., benzodiazepines, antihypertensives (ATC C09, C08, C07, C03, C02), and anticholinergics (urological, antidepressant, other)], and alcohol. OR, Odds ratios; CI, confidence intervals; *p, p*-value of binomial logistic regression result; P_H−L_, *p*-value of Hosmer–Lemeshow test of goodness of fit; P_L−R_, *p*-value of likelihood ratio test; M1:M2, likelihood ratio test conducted between model 1 and model 2; M2:M3, likelihood ratio test conducted between model 2 and model 3.

To assess the effects of musculoskeletal conditions on the development of depression, binomial logistic regression was conducted to quantify the associations (i.e., ORs) between the musculoskeletal conditions and probable LLD at baseline and Wave 2 (Figure 1).

For the cross-sectional studies, the prevalence of probable LLD was 8.55% (*n* = 429). Among all of the musculoskeletal conditions, arthritis, osteoporosis, and MCP were confirmed to be significantly associated with probable LLD. The crude model attested that arthritis, osteoporosis, and MCP were associated with 76.2% (OR = 1.762, 95% CI: 1.434–2.160; *p* < 0.001), 55.4% (OR = 1.554, 95% CI: 1.158–2.055; *p* = 0.003), and 195.1% (OR = 2.951, 95% CI: 2.412–3.618; *p* < 0.001) higher odds of probable LLD. Sensitivity analyses further substantiated the robustness of these associations. The OR, as the quantitative indicator of associations between arthritis, osteoporosis, MCP, and depression, fluctuated a little but was generally stable (Figure 1).

For the longitudinal studies, the prevalence of probable LLD was 4.92% (*n* = 226). As we expected, arthritis, osteoporosis, and MCP were reaffirmed to be significantly associated with probable LLD. Arthritis, osteoporosis, and MCP were associated with 123.3% (OR = 2.233, 95% CI: 1.700–2.927; *p* < 0.001), 68.1% (OR = 1.681, 95% CI: 1.133–2.421; *p* = 0.007), and 140.4% (OR = 2.404, 95% CI: 1.838–3.151; *p* < 0.001) higher odds of probable LLD in the crude model. In further sensitivity analyses, the robustness of these associations was confirmed (Figure 1).

Comprehensively considering the results of the goodness-of-fit test and the likelihood ratio test (Figure 1), model 1, model 2, and model 3 were deemed different during the sensitivity analyses. Thus, the robustness of the associations between arthritis, osteoporosis, MCP, and LLD was validated using sensitivity analyses (44).

### Arthritis to LLD

**Figure 2:**
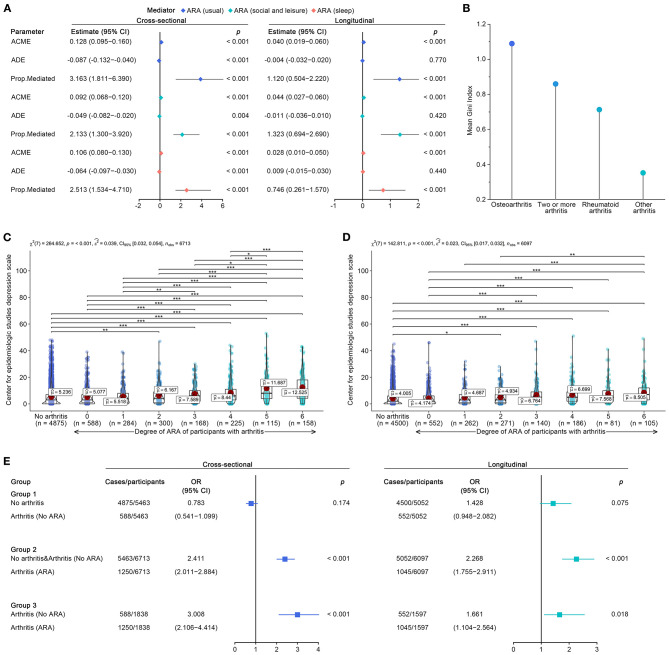
Further analyses for restriction of activities, and subtypes in arthritis. **(A)** Results of cross-sectional and longitudinal mediation analysis for arthritis, in which ARA (usual), ARA (social and leisure), and ARA (sleep) were considered as potential mediators. **(B)** Random-Forest algorithm-derived Mean Gini Index weighed the contribution of arthritis subtypes to outcomes of depression. **(C)** Cross-sectional potential difference of depressive symptoms among participants with/without arthritis (stratified by degree of ARA). **(D)** Longitudinal potential difference of depressive symptoms among participants with/without arthritis (stratified by degree of ARA). **(E)** Stratified analyses using binomial logistic regression quantified associations between arthritis (No ARA), arthritis (ARA), and depression at baseline and Wave 2. ARA, Restriction of activities caused by arthritis; ACME, average causal mediation effects; ADE, average direct effects; Prop. mediated, proportion mediated; χ^2^, chi-square; n_obs_, number of observation; û, group mean; $\hat{\varepsilon^{2}},\;$effect size of ε^2^; *p, p*-value. *p*-value adjusted < 0.05 was represented by “*,” *p*-value adjusted < 0.01 was represented by “**,” *p*-value adjusted < 0.001 was represented by “***”.

To determine whether the arthritis-induced restriction of activities (ARA) explained the increased depression risk, we tested a series of mediation models. Interestingly, our results were somewhat contrary to the results observed in other works, which simply concluded that arthritis was significantly linked to enhanced risks for depression (45, 46); however, the causal pathways between arthritis and depression are far from simple. The present results newly discovered and elaborated one of the potential causal pathways between arthritis and depression, as shown in Figure 2A. In cross-sectional mediation analyses, the direct effects of arthritis attenuated the risk for depression, while ARAs presented completed mediation effects that mediated 316.3% (ARA of usual), 213.3% (ARA of social and leisure), and 251.3% (ARA of sleep). The results of the longitudinal studies were very similar. Arthritis-induced direct effects attenuated or did not affect the depression risk, and ARAs demonstrated mediation effects that mediated 112.0% (ARA of usual), 132.3% (ARA of social and leisure), and 74.6% (ARA of sleep). Further analyses of potential differences in depressive symptoms corroborated the results of the mediation analyses. No statistically significant difference was detected between participants without arthritis and arthritis participants without ARA. In contrast, depressive symptoms gradually increased with the increasing degree of ARA (*p* < 0.001, middle effect sizes), regardless of the cross-sectional (Figure 2C) or longitudinal design (Figure 2D). Stratified analyses using binomial logistic regression reaffirmed that no significant association existed between arthritis without ARA and probable depression (Figure 2E). Considering that the participants without arthritis and the arthritis participants without ARA had no significant difference in depressive symptoms and that their group means were similar, the two were classified together for further stratified analyses. Arthritis with ARA was associated with 141.1% (OR = 2.411, 95% CI: 2.011–2.884 for cross-sectional) and 126.8% (OR = 2.268, 95% CI: 1.755–2.911 for longitudinal) higher odds of probable depression (vs. no arthritis and arthritis without ARA). When further restricted to participants with arthritis, arthritis with ARA was associated with 200.8% (OR = 3.008, 95% CI: 2.106–4.414 for cross-sectional) and 66.1% (OR = 1.661, 95% CI: 1.104–2.564 for longitudinal) higher odds of probable depression.

Subsequently, the contributions of arthritis subtypes to depressive symptoms were investigated in Wave 2. As a result of the random forest displayed in Figure 2B, osteoarthritis contributed the most to the increased depression risk, followed by two or more arthritis, rheumatoid arthritis, and other arthritis (the mean Gini index was 1.089, 0.860, 0.713, and 0.352 for longitudinal studies).

### Osteoporosis to LLD

**Figure 3:**
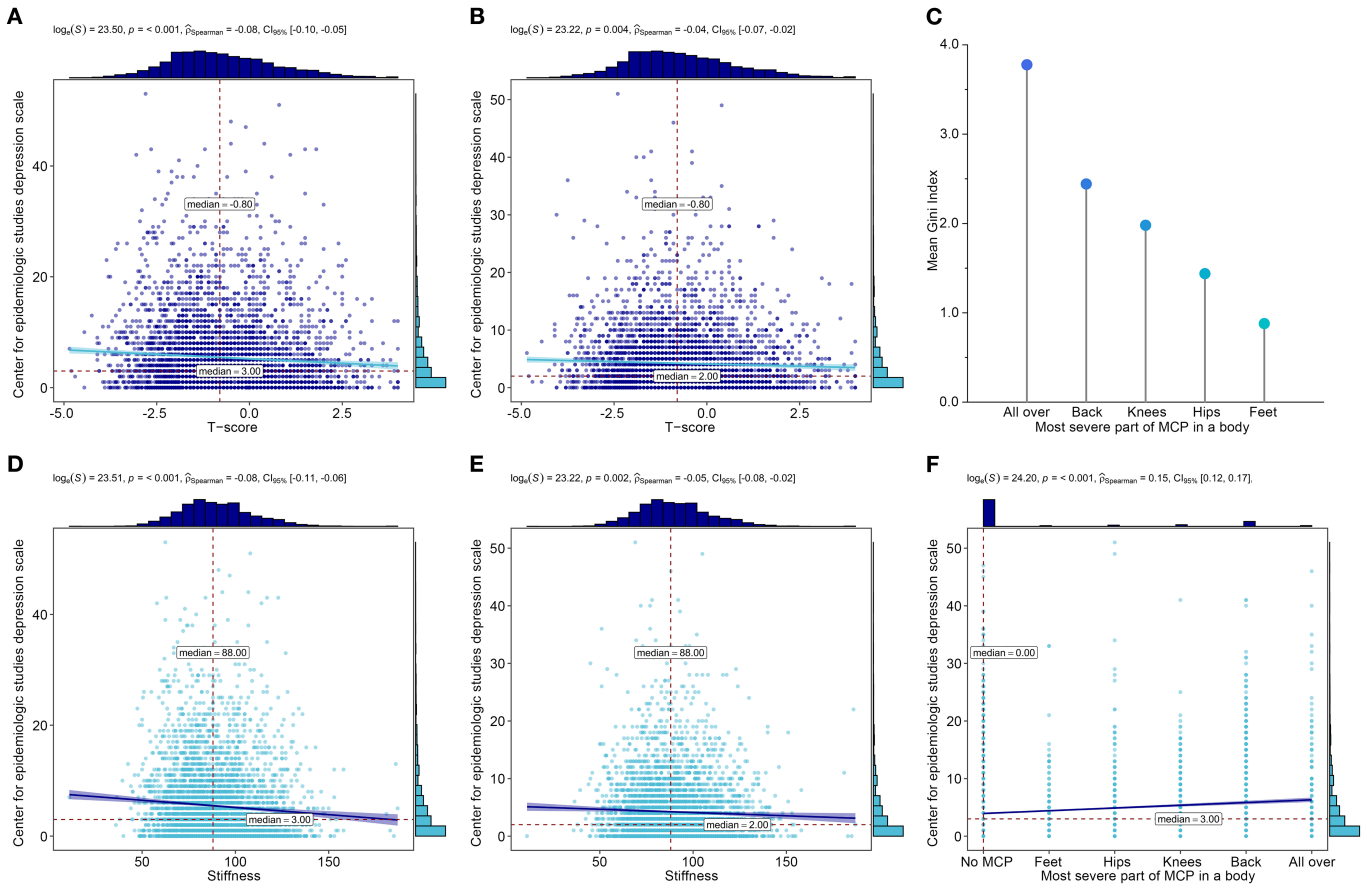
Further analyses for osteoporosis and MCP. **(A)** Association between *T*-score and depressive symptoms at baseline**. (B)** Association between *T*-score and depressive symptoms at Wave 2. **(C)** Mean Gini Index quantified the contributions of different body parts with the most severe MCP. **(D)** Association between stiffness and depressive symptoms at baseline. **(E)** Association between stiffness and depressive symptoms at Wave 2. **(F)** Association between the most severe body part of MCP and depressive symptoms at Wave 2.

Fragility fractures are the most common and severe complications of osteoporosis (47–49); therefore, we tested whether they mediated the increased risk for LLD. Mediation analysis found that osteoporosis-associated fractures that newly occurred at Wave 2 did not mediate the association between osteoporosis and LLD (Supplementary Table 1). *T*-score and stiffness as indicators for osteoporosis were negatively correlated with depressive symptoms, regardless of baseline (Figures 3A,D) or Wave 2 (Figures 3B,E).

### MCP to LLD

We investigated whether the difference in the most severe body part of MCP affects the increased risk for LLD at Wave 2. As a result of the random forest presented in Figure 3C, systemic pain contributed the most to the increased risk for LLD, followed by back, knees, hips, and feet (the mean Gini index was 3.778, 2.442, 1.980, 1.438, and 0.879, respectively). It was significantly associated with depressive symptoms (Figure 3F).

### Potential Difference of Depressive Symptoms at Wave 2

As we expected, participants with arthritis, osteoporosis, or MCP tended to report more severe depressive symptoms (arthritis: *p* < 0.001, $\hat{r}$ = 0.109, 95% CI: 0.082–0.130, small effect size; osteoporosis: *p* = 0.010, $\hat{r}$ = 0.040, 95% CI: 0.014–0.068, small effect size; MCP: *p* < 0.001, $\hat{r}$ = 0.169, 95% CI: 0.146–0.194, small effect size) (Supplementary Figure 2). Among the three disorders, females were always more likely to report more severe depressive symptoms than males (Supplementary Figure 3). These resultant data indicate that gender-related differences in depressive symptoms were non-negligible.

### IMC-Induced Negative Psychological Risk Classification Approach

**Figure 4:**
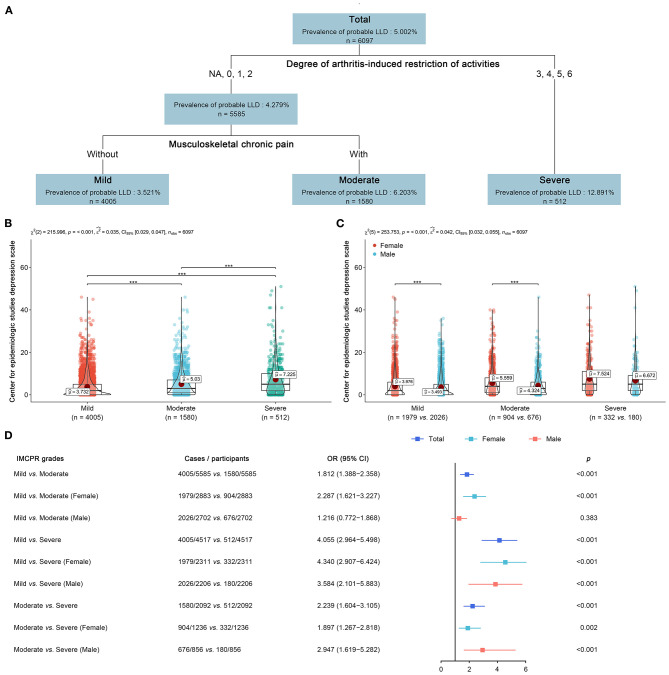
IMC-induced negative psychological risk classification approach. **(A)** IMCPR classification approach was derived using tree regression**. (B)** Difference of depressive symptoms among participants with different IMCPR grades. **(C)** Potential gender-related difference of depressive symptoms among participants with different IMCPR grades. **(D)** Stratified analyses using binomial logistic regression derived odds ratios (OR) and 95% confidence intervals (CI) as indicators of association between IMCPR grades and depressive disorder, in overall, female, or male cohort. NA, No arthritis.

Musculoskeletal disorders (arthritis, osteoporosis, and MCP) significantly associated with LLD were included for decision tree regression. The derived IMCPR classification approach, including arthritis and MCP, could stratify older people into three grades of mild, moderate, and severe (Figure 4A). Details of the IMCPR classification approach are given in Supplementary Table 2. Within the tree regression analysis of the overall cohort, the most significant factor conveying risk for LLD was arthritis. The prevalence of probable LLD was 3.521% (mild), 6.203% (moderate), and 12.891% (severe). Analyses for depressive symptoms reported significant differences among the three grades (*p* < 0.001, $\hat{\varepsilon^{2}}$ = 0.035, 95% CI: 0.029–0.047, small effect size) (Figure 4B), and the significant difference remained when further considering gender (*p* < 0.001, $\hat{\varepsilon^{2}}\;=$ 0.042, 95% CI: 0.032–0.055, small effect size) (Figure 4C). Of particular note, females always tended to report more severe depressive symptoms than males. Overall, participants who were classified as “severe” had 305.5%/123.9% higher odds of LLD than “mild”/“moderate” and “moderate” participants, who possessed 81.2% higher odds of LLD than “mild” participants (Figure 4D). Gender differences must be recognized. The results of the stratified analyses for females agreed with this tendency of OR distribution, but for males, it partly did not (Figure 4D). Males had higher odds of LLD only when they were graded as “severe.” Specifically, females who were classified as “severe” had higher odds of depression by 334.0% relative to those classified as “mild,” while males who were classified as “severe” only had higher odds of depression by 258.4% relative to those classified as “mild”.

## Discussion

This prospective study on data from a population–representative sample of older people living in the same context represents, to our knowledge, the first attempt to systematically investigate whether musculoskeletal conditions and surgery are associated with increased risks for negative psychological health outcomes and to further explore the underlying mediators. It is also the first large longitudinal study using machine learning that sought to derive a classification approach to assess the potential augmented psychiatric risks of older people who suffered from musculoskeletal disorders.

We investigated not only whether but also how individual musculoskeletal conditions that commonly occur in older people, such as arthritis, osteoporosis, fracture, joint replacement operations, and MCP, could confer an enhanced risk for LLD. One important finding revealed by the present study was that it is not the original arthritis itself but rather the arthritis-induced restriction of individual activities that plays a prominent role in the development of psychiatric disorders in these elderly populations. The present results updated the recognition generalized in other works, which simply deemed arthritis as a hazardous factor resulting in depression (45, 46); however, the causal pathways between arthritis and depression were underappreciated. The resultant data found that arthritis did not convey a risk for negative psychological health outcomes unless it restricted individual activities (usual, social and leisure, or sleep). Arthritis sufferers with ARA augmented the odds of probable LLD after 2 years by 126.8%, while arthritis sufferers without ARA were not compromised by such risks. Restriction of activities completely mediated the association between arthritis and depression (ARA: 316.3% of usual, 213.3% of social and leisure, and 251.3% of sleep), and depressive symptoms gradually augmented with the escalating degree of ARA.

In the elderly subjects, the findings of arthritis collected in the present study are in line with previous work (50–52), demonstrating that arthritis may contribute to depressive disorders/symptoms over time. The present study provided further supporting evidence to clinicians who may transfer the focus from arthritis itself to the arthritis-induced restriction of activities as a significant trigger for medical interventions among elderly sufferers. The assessment scale of the degree of ARA is enclosed in this study for potential medical applications. Moreover, rheumatoid arthritis is generally considered a critical factor in the development of depression in the reported studies (45, 53, 54), which is contrary to our findings. The random forest algorithm confirmed that among the subtypes of arthritis, osteoarthritis contributed the most to the increased risk for LLD (the Gini Index was 1.089 for longitudinal studies), which may be explained by osteoarthritis being a common and disabling condition that affects the daily functioning and independence of older people (55, 56). It was reported that osteoarthritis, as the most prevalent subtype of arthritis in older people, contributes more to YLD than rheumatoid arthritis (4). Obviously, osteoarthritis and its underlying mechanisms leading to an increased risk for psychological disorders warrant further investigation.

Our findings highlight the importance of arthritis-induced restriction of activities, which completely mediated the association between arthritis and depression. Activity restrictions are strongly associated with depression in older people (57). Unfortunately, arthritis is always inseparable from the restriction of activities (56). Given the high incidence of arthritis in older people, it is remarkably important to emphasize the symptoms that can actually lead to depression.

Osteoporosis and MCP often convey a risk for depressive disorder (58, 59), which was corroborated in the present study on population–representative samples. In addition, we found that osteoporosis-related fracture, as the most common complication of osteoporosis, did not mediate the enhanced risk for LLD. More importantly, it is noteworthy that such severe, acute trauma should not be considered a potential risk factor for LLD according to our findings. *T*-score and stiffness, as diagnostic clinical parameters for osteopenia and osteoporosis (60), were negatively associated with depressive symptoms. This result indicated that depressive symptoms were augmented with the increasing severity of osteoporosis (decreased bone mineral density), which could serve as a potential indicator for increased risk for LLD in the development of osteoporosis. For MCP, we attested that different severe body parts of MCP convey different degrees of depressive symptoms, which might be conducive to guiding stratified interventions for older people with MCP.

The most important aspect of the present study lies in the IMC-induced negative psychological risk classification approach for the prediction and quantification of increased risks for depression. The IMCPR classification approach was derived by decision tree regression from all musculoskeletal disorders that were confirmed to be associated with an augmented risk for probable LLD in our study. Our results demonstrated that older people with a higher grade of IMCPR suffered an augmented risk for worse psychological health outcomes. In detail, a patient with a grade of “severe” had 305.5/123.9% higher odds of depression than those with a “mild”/“moderate” grade.

These findings might offer insight into the clinical treatments of older people who suffer from musculoskeletal disorders and highlight the importance of refined stratified psychological interventions to ensure the quality of life of the older population. Older people classified at lower grades of IMCPR would not require excessive psychological intervention, while those classified as higher grades demand comprehensive interventions. Musculoskeletal disorders, which generally compromise older people, exacerbate the global disease burden and consume enormous socioeconomic costs (4, 14). Aided by our IMCPR classification approach, older people with higher odds of LLD could be regularly identified; such a classification approach could not only reduce unnecessary psychological interventions but also potentialize precision medicine and optimize the redistribution of social-medical resources.

Gender-related differences in depressive disorder/symptoms were also taken into consideration in the present study. Among musculoskeletal disorders, females always tend to report higher odds of depression or poorer depressive symptoms than males. Such gender-related differences remained in the IMCPR classification. Females had two- to three-fold higher rates of depression than males, as previously reported (61), of which several explanations have been offered: (1) Sexual dimorphism at the transcriptional level mediates gender-specific stress susceptibility (62). (2) Gender-related differences in neurotransmitter, endocrine, and metabolic systems result in differences in mood susceptibility (61). Our results from the stratified analyses revealed that, as we hypothesized, females had higher odds of probable LLD than males in the older population who were classified at the same IMCPR grade. Females with a “severe” grade had higher odds of LLD by 334.0% relative to those with a “mild” grade, while males had higher odds of LLD by only 258.4%. The proposed IMCPR classification approach considering gender-related differences has huge potential for clinical application because it was confirmed as outstanding for negative psychological risk stratification referring to different genders.

We acknowledge that this study has limitations. Chiefly, limited to the methods and original data of large-scale cohort research (TILDA), both activity restriction and the IMCPR classification approach are based on self-reported symptoms, and the present IMCPR classification approach demands further validation in clinical practice as already planned. For the same reason, we could not evaluate the effects of frailty score, mobility scores, step count, and vitamin D status. These indicators will be adopted in the follow-up studies after further optimized design with the inclusion of more extensive and detailed source data. Furthermore, the genetic mechanism of ARA-mediated effects requires further experimental research.

Overall, the present study, based on machine learning algorithms and various models, including a mediation model, discovered and elaborated the role and the underlying associations of musculoskeletal conditions in the development of depression in the elderly population and further fostered a novel IMCPR approach for depressive risk stratification. We found that arthritis does not convey a risk for negative psychological health outcomes unless it restricts individual activities (usual, social and leisure, or sleep). Furthermore, osteoarthritis, not rheumatoid arthritis, contributes the most to depressive symptoms. Among the musculoskeletal disorders, arthritis with activity restriction was the leading contributor to depression. Bone mineral density was negatively associated with depressive symptoms, and the depressive symptoms varied with the body part with the most severe MCP. The most important finding is that the IMCPR classification approach that we developed could specifically identify older people who require psychological intervention, thus optimizing social–medical resource redistribution and refining precision medicine by reducing the related global medical consumption due to musculoskeletal diseases.

## Data Availability Statement

The original contributions presented in the study are included in the article/Supplementary Material, further inquiries can be directed to the corresponding author.

## Ethics Statement

The studies involving human participants were reviewed and approved by Trinity College Dublin Research Ethics Committee. The patients/participants provided their written informed consent to participate in this study.

## Author Contributions

WJ: conceptualization and visualization. WJ and ZL: methodology and writing—original draft preparation. WJ, ZL, and YZ: data curation. WJ, ZL, and ZC: formal analysis. MG: writing—review and editing, project administration, and funding acquisition. All authors contributed to the article and approved the submitted version.

## Conflict of Interest

The authors declare that the research was conducted in the absence of any commercial or financial relationships that could be construed as a potential conflict of interest.

## Publisher's Note

All claims expressed in this article are solely those of the authors and do not necessarily represent those of their affiliated organizations, or those of the publisher, the editors and the reviewers. Any product that may be evaluated in this article, or claim that may be made by its manufacturer, is not guaranteed or endorsed by the publisher.

## Abbreviations

IMC: Individual musculoskeletal conditions
IMCPR: IMC-induced negative psychological risk
ARA: arthritis-induced restrictions of activities
YLDs: years lived with disability
MCP: musculoskeletal chronic pain
TILDA: The Irish Longitudinal Study on Aging
CES-D: Center for Epidemiological Studies Depression Scale
LLD: late-life depression
TG: triglyceride
LDL: low-density lipoprotein cholesterol
HDL: high-density lipoprotein cholesterol
CHOL: total cholesterol
BMI: body mass index
OR: odds ratios
CI: confidence intervals
P_H−L_: *p*-value of Hosmer–Lemeshow test of goodness of fit
P_L−R_: *p*-value of likelihood ratio test
M1:M2: likelihood ratio test conducted between model 1 and model 2
M2:M3: likelihood ratio test conducted between model 2 and model 3
ACME: average causal mediation effects
ADE: average direct effects
Prop. mediated: proportion mediated
χ^2^: chi-square
n_obs_: number of observation
û: group mean
$\hat{\varepsilon^{2}}$: effect size of ε^2^
*p*: *p*-value.
