# Supplementary material for: The Effect of Individual Musculoskeletal Conditions on Depression: Updated Insights From an Irish Longitudinal Study on Aging
Source: Front Med (Lausanne). 2021 Aug 26;8:697649. doi: 10.3389/fmed.2021.697649 (PMC8426633; doi:10.3389/fmed.2021.697649)

**Supplemental material**

**Table. S1.** Mediation analysis for osteoporosis, in which the osteoporosis-associated pathological fracture newly occurred at Wave 2 was considered as a potential mediator.

**Table. S2.** Detail of IMCPR classification approach.

**Figure. S1.** Details about study design and participants.

**Figure. S2.** Potential difference of depressive symptoms at Wave 2.

**Figure. S3.** Gender-related potential difference of depressive symptoms at Wave 2.

**Table. S1.** Mediation analysis for osteoporosis, in which the osteoporosis-associated pathological fracture newly occurred at Wave 2 was considered as a potential mediator.

| **Parameters** | **Estimate (95% CI)** | ***p*** |
| --- | --- | --- |
| **ACME** | -0.003 (-0.014－0.010) | 0.634 |
| **ADE** | 0.028 (0.004－0.060) | 0.022 |
| **Prop. Mediated** | -0.100 (-1.379－0.450) | 0.636 |

Abbreviations: Average causal mediation effects (ACME); average direct effects (ADE); proportion mediated (Prop. mediated).

**Table. S2.** Detail of IMCPR classification approach.

| **Scale for degree of ARA** | | | |
| --- | --- | --- | --- |
| **Question 1** | | | |
| *Does your arthritis make it difficult for you to do your usual activities such as household chores or work?* | | | |
| **Question 2** | | | |
| *Does the arthritis limit your social and leisure activities?* | | | |
| **Question 3** | | | |
| *Does your arthritis make it difficult for you to sleep at night?* | | | |
| **Options:** | No | Sometimes | Yes |
| **Score:** | 0 | 1 | 2 |
| Calculating the accumulative scores of options of 3 questions, the final score is the degree of ARA. | | | |
|  |  |  |  |
| **IMCPR classification approach** | | | |
| **Musculoskeletal status** | | | **Grade** |
| Without arthritis or the degree of ARA ranged from 0 to 2, without MCP. | | | Mild |
| Without arthritis or the degree of ARA ranged from 0 to 2, with MCP. | | | Moderate |
| The degree of RA ranged from 3 to 6, with/without MCP. | | | Severe |

Abbreviations: Arthritis-induced restriction of activities (ARA); musculoskeletal chronic pain (MCP).

**Figure. S1.** Details about study design and participants.


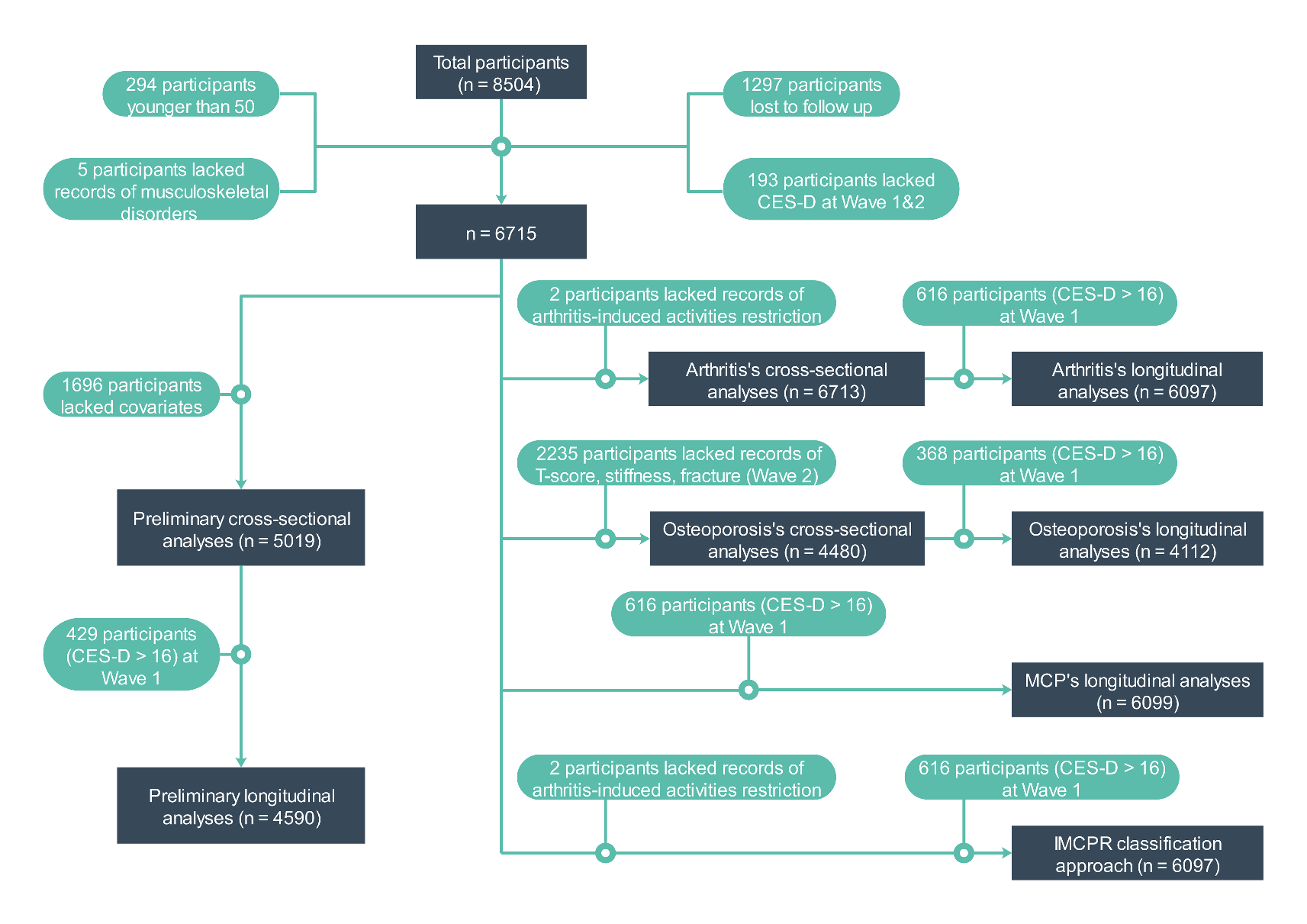


**Figure. S2.** Potential difference of depressive symptoms at Wave 2.

Potential difference in depressive symptoms of arthritis (A), osteoporosis (B), MCP (C) were investigated in the corresponding cohort.


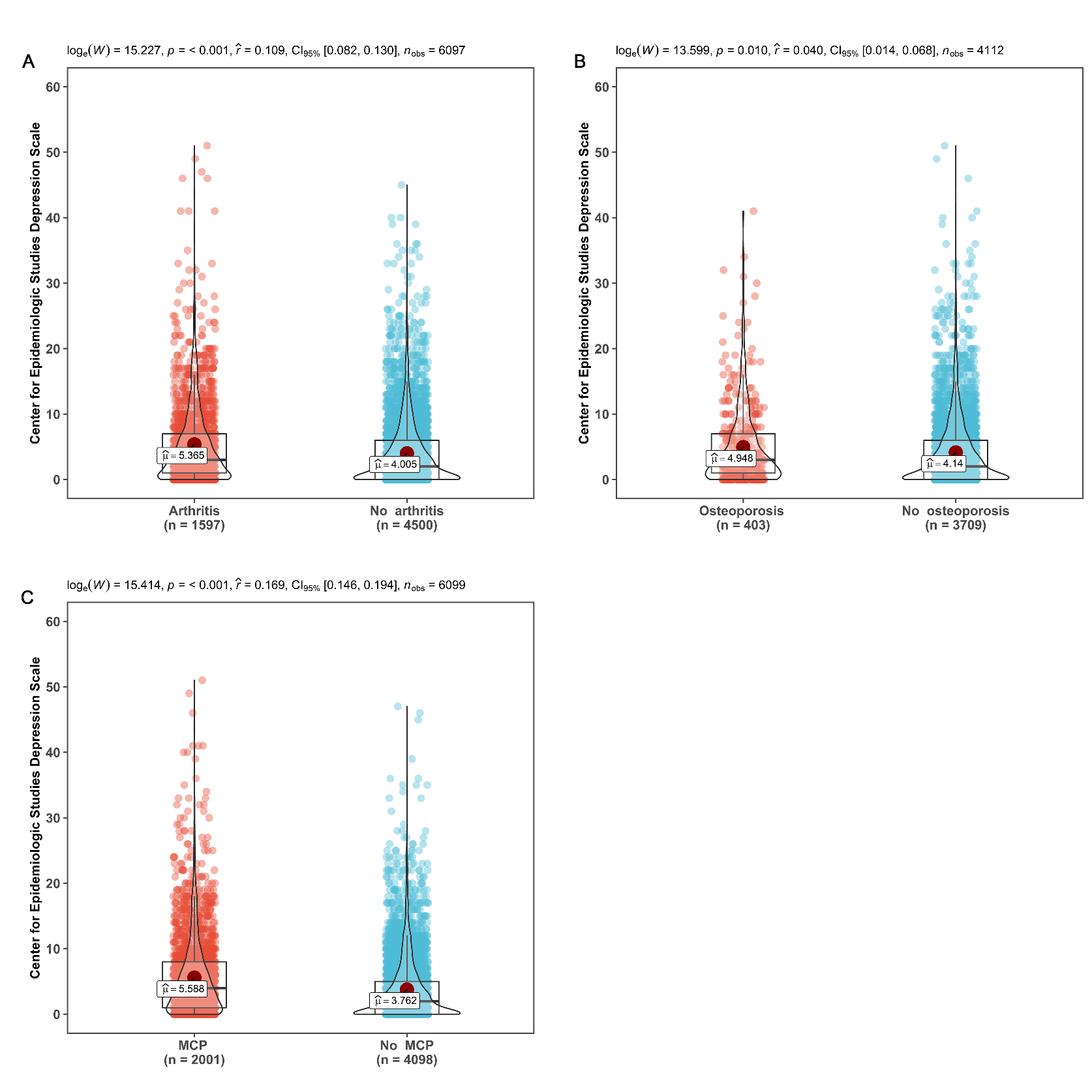


**Figure. S3.** Gender-related potential difference of depressive symptoms at Wave 2.

Gender-related potential difference in depressive symptoms of arthritis (A), osteoporosis (B), MCP (C) were investigated in the corresponding cohort.


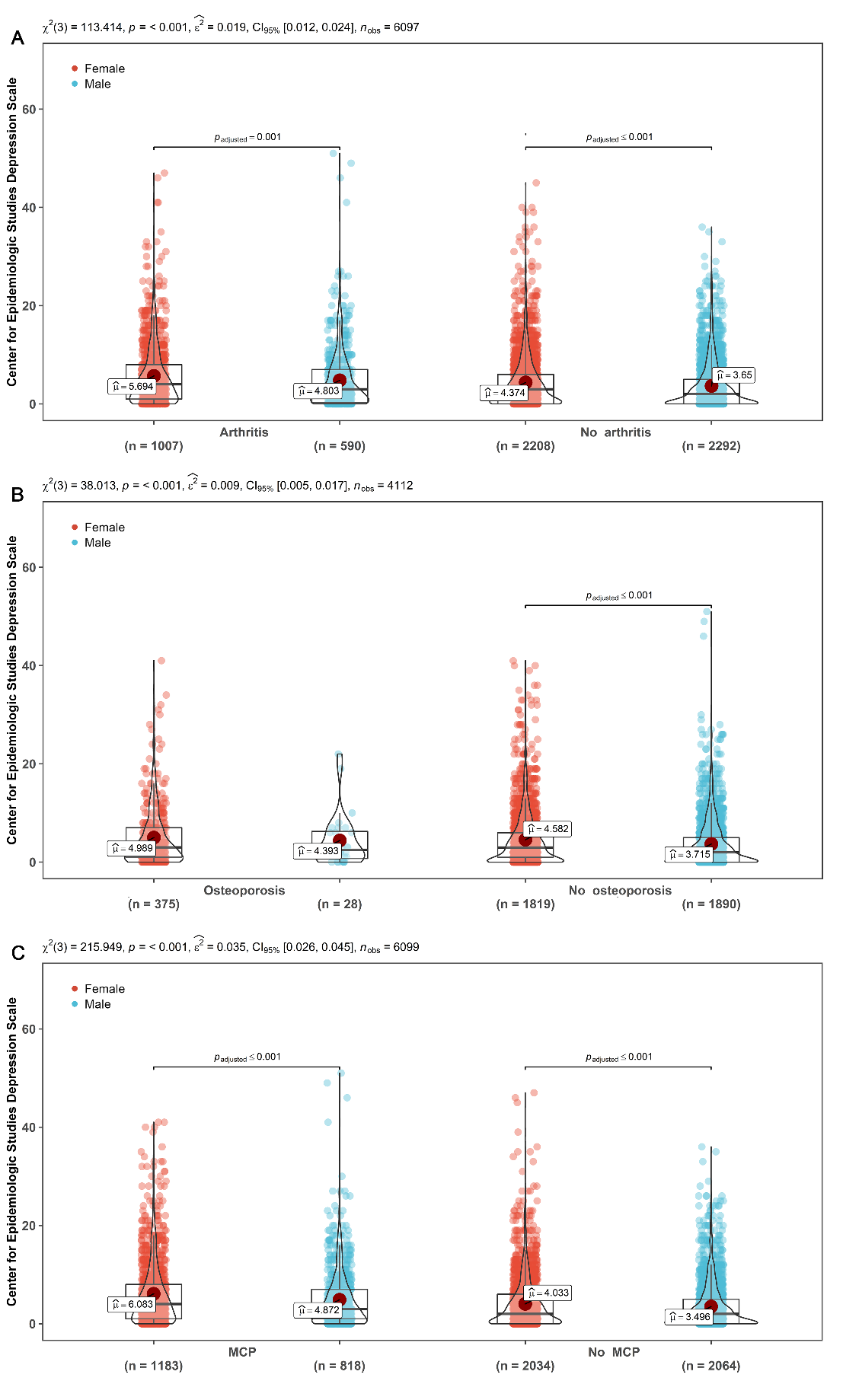

Supplement: Supplementary file 1 [file Data_Sheet_1.DOCX]
